# Microscale Symmetrical Electroporator Array as a Versatile Molecular Delivery System

**DOI:** 10.1038/srep44757

**Published:** 2017-03-20

**Authors:** Mengxing Ouyang, Winfield Hill, Jung Hyun Lee, Soojung Claire Hur

**Affiliations:** 1 Rowland Institute at Harvard University, 100 Edwin H. Land Blvd., Cambridge, MA 02142, USA; 2 Massachusetts General Hospital, Charlestown, MA 02129, USA

## Abstract

Successful developments of new therapeutic strategies often rely on the ability to deliver exogenous molecules into cytosol. We have developed a versatile on-chip vortex-assisted electroporation system, engineered to conduct sequential intracellular delivery of multiple molecules into various cell types at low voltage in a dosage-controlled manner. Micro-patterned planar electrodes permit substantial reduction in operational voltages and seamless integration with an existing microfluidic technology. Equipped with real-time process visualization functionality, the system enables on-chip optimization of electroporation parameters for cells with varying properties. Moreover, the system’s dosage control and multi-molecular delivery capabilities facilitate intracellular delivery of various molecules as a single agent or in combination and its utility in biological research has been demonstrated by conducting RNA interference assays. We envision the system to be a powerful tool, aiding a wide range of applications, requiring single-cell level co-administrations of multiple molecules with controlled dosages.

The delivery of macromolecules such as protein^1^,^2^, DNA^3^,^4^ and RNA^5^,^6^ into cells is a crucial but challenging task for biological and clinical research to elucidate the role of these molecules in regulating cellular functions. Particularly, intracellular co-delivery of multiple molecules would enable identification of optimum combinatorial molecular ratios for various applications, including drug screening for combination therapy^7^,^8^,^9^ and cellular reprogramming, utilizing multiple transduction factors^10^,^11^,^12^. The main hurdle of intracellular macromolecule delivery lies with the difficulty in simultaneously achieving high transfection efficiency, prolonged efficacy, and low cytotoxicity for a wide range of molecules. Among prevalent delivery methods, viral-mediated gene delivery provides predominant efficiency; however, it is limited for nucleic acid delivery and retains safety concerns associated with immunogenicity^13^ and viral genome integration^11^. Consequently, it is inapplicable for recent combinatorial therapeutic approaches utilizing co-delivery of genetic and other therapeutic materials^14^. Chemical-mediated exogenous molecular delivery, on the other hand, is suitable for limited applications due to its impractical efficiency. An attractive alternative method for multi-molecular delivery is electroporation because of its capability to introduce countless types of molecules into cells via transiently formed pores on cellular membranes upon exposure to short high-voltage pulses^15^. However, conventional electroporation^12^,^16^,^17^ is unsatisfactory for fragile cells because of high mortality and cytolysis rate associated with inevitably high operational voltages (≥200 V) required to obtain practical efficiency. Recent advances in microfluidic-based electroporation systems^18^,^19^,^20^,^21^,^22^,^23^ operating at lower voltages enable enhancement in efficiency and viability, even providing single-cell level molecular delivery. Challenges in dose-controlled multi-molecular delivery into fragile cells, however, still remain because of their limited throughput and/or single-directional flow scheme employed in such systems.

To address these limitations, we developed a robust, efficient and versatile on-chip vortex-assisted electroporation system, namely Microscale Symmetrical Electroporator Array (μSEA), enabling simultaneous target cell enrichment and multi-molecular delivery in one integrated process (Fig. 1). By utilizing the vortex-assisted electroporation method^24^,^25^, μSEA provides benefits, including real-time visualization of the process, precise dosage control, uniform cytosol distributions of delivered molecules, multi-molecular delivery with high efficiency and viability. Even though μSEA shares the core vortex-assisted electroporation concept with its predecessors^24^,^25^, extensive modeling and empirical iterations implemented for the μSEA’s electrode design offers unprecedented design flexibility and integrability. It additionally provides differentiated benefits such as substantial operational voltage reduction (V_applied_ < 20 V), and most importantly, enhanced electroporation efficiency and cell viability. In addition, the sequential multi-molecular delivery capability was expanded to the co-delivery of three types of macromolecules accompanied with quantitative analyses, which has rarely been reported to the best of our knowledge. The versatility and robustness of μSEA is further evidenced by efficient delivery of a wide range of biomolecules, including fluorescent dyes, proteins, siRNA and miRNA, into various cell types. By taking advantage of the high-throughput rare cell purification capability that the adopted Vortex chip’s chamber geometry provides^26^, the current system has great potential to extend applications of electroporation directly to cells purified from complex bodily fluids.

## Results and Discussion

### Device Design

μSEA is composed of a vortex cell trapping chamber array enclosed by a glass slide with micro-patterned Au electrodes, generating sufficient electric fields for electroporation on orbiting cells. Dimensions and arrangements of micro-patterned electrodes are optimized to minimize undesired voltage drops across the connecting electrodes and the voltage variation across chambers while ensuring irreversible sealing between the PDMS layer and the glass substrate. Extensive COMSOL and Simulation Program with Integrated Circuit Emphasis (SPICE) simulations were conducted to predict the optimum electrode design and to estimate initial electric parameters to be experimentally tested. Various microelectrodes and layout designs (Table S1, Supplementary Information) were evaluated and optimized using SPICE simulation by modeling the electrode network as a complex circuit (Figure S1, Supplementary Information). In general, increase in the number of electroporation chambers and rows lead to larger voltage variations due to the increased complexity of electrode layout. Thus, the goal of optimization was to maximize the voltage efficiency, V_eff_, and to minimize the voltage variation across chambers, ΔV_C_. Here, V_eff_ is the ratio between the voltage measured across the electroporation array and the actual voltage applied, V_input_, while ΔV_C_ is the maximum voltage differences among all electroporation chambers. The staggered chamber design (Design 1 in Table S1) permitted to arrange higher number of chambers in a given field of view, thus increasing overall throughput (22 chambers in a given footprint); however, it resulted in undesirable lengthening of electrodes connecting the interdigitated electrodes in the chambers to the common bus line, yielding dramatically increased ΔV_C_. On the other hand, the vertically aligned chamber arrangement (Design 2 in Table S1) lowered the overall throughput (16 chambers in a given footprint), but it exhibited increased V_eff_ with reduced ΔV_C_. Accordingly, the arrangement of electrodes and chambers for μSEA (Design 3 in Table S1) was built upon that of Design 2 while enhancing the overall throughput by parallelizing more channels and attaining competitive V_eff_ by shortening distances between chambers (40 chambers per device, detailed device geometry in Methods). μSEA achieved V_eff_ = 80% and ΔV_C_ < 5% with 10-fold lower operational voltage and 4-fold increase in throughput from the previous vortex-assisted electroporation system^24^,^25^. The voltage distribution and electric field intensity inside a single chamber were computed using COMSOL (Figure S2, Supplementary Information) to predict the voltage range that surpasses the electric field threshold (E > 0.6 kV/cm)^24^, as required for successful vortex-assisted electroporation. In addition, micro-patterned Au electrodes could be seamlessly integrated with microfabrication process flows, allowing batch production at low cost.

### Electroporation Parameter Optimization

Our previous works, consisting of a single-24 and 10-chambers^25^, utilized a pair of bulk Aluminum wires (1 mm in diameter) across each chamber, and relatively high voltage pulses (≥100 V) generated using a direct current (DC) power source were required to achieve optimal electroporation results. In contrast, the current 40-chamber μSEA, composed of microscale planar electrodes operating with alternating current (AC) electric fields to minimize the disadvantages of utilizing DC pulses, including inevitable bubble formation and pH changes^27^,^28^, was able to provide more gentle and controllable membrane permeation process.

The electroporation performance evaluations revealed AC square wave pulses at 20 kHz as the optimum for the current configuration. Square waveform was chosen over sine waveform because square waveform required simpler design for voltage source electronic circuitry than its counterpart and it performed slightly better when the identical peak AC voltage was applied (Figure S3, Supplementary Information). Lower frequencies (f ≤ 10 kHz) induced unwanted electrolysis that interferes with cell-trapping vortices and causes electrode damage while higher frequency (f ≥ 40 kHz) yielded degraded efficiency due to overquick shifts between electric polarities. Although the optimum voltage for successful electroporation varied slightly depending on cell types, the real-time visualization of the fluorescent traces of electroporated cells orbiting in vortices enabled prompt modifications of electroporation parameters (i.e., voltage, frequency, and pulse width) and incubation duration (Fig. 2a). This implies that the versatility and flexibility of μSEA allow electroporation of cells from unknown origins with no prior information. The standard electrical parameters used for all the experiments described in this manuscript were set as square wave pulses with frequency, f = 20 kHz, pulse width, τ = 1 ms, and pulse interval, Δt = 1 s, unless stated otherwise. The optimum voltages for MCF7, HeLa and both HEK293 and MDA-MB-231 were found to be 17 V (1.02 kV/cm), 16 V (0.96 kV/cm) and 15 V (0.90 kV/cm), respectively (Fig. 2b–e). The corresponding electric field intensities were computed via COMSOL simulations and the values represent the average electric field intensities estimated at 20 μm above the electrode surface where the trapped cell orbits, exposed to the highest electric fields are located. At voltages greater than the optimum value, the efficiency remained high but the total number of electroporated cells collected off-chip decreased, presumably caused by bursting of cells with larger diameters. This phenomenon can be strategically utilized for various applications, preferably requiring transfected cells with higher purity and reduced cell density in single-cell resolutions^29^,^30^,^31^.

### Dosage Control and Multi-Molecular Delivery

By eliminating laborious sequential pipetting processes or undesired cell loss in-between treatments^24^,^25^, μSEA’s sequential multi-molecular delivery capability would enable the performing of various assays that require combinational administrations of multiple substances^32^,^33^. μSEA’s rapid multi-solution exchange scheme allows precise control of delivery dosages of individual molecules, and the ease of response evaluations for cells exposed to a wide range of molecular administration ratios. We have previously demonstrated that incubating trapped cells for varied durations allowed for the delivery of varying doses of small molecules, such as fluorophores and chemotherapeutic drugs as single agents or in combination^25^. Here, a similar approach has been utilized to deliver proteins and siRNA. Quantitative analyses of fluorescent intensities of electroporated cells revealed that the amount of delivered proteins and siRNA increased with incubation durations (Figs 2f and 3). The wider fluorescent intensity distribution for electroporated cells incubated in a GFP solution for 120 s might be originated from the saturation of the molecular uptake or resealing of the cell membrane.

To evaluate sequential delivery capability of μSEA for macromolecules that are considered to be more difficult to deliver, three types of proteins with various molecular weights (27k–66kDa) and structures were injected into the electroporated HEK293 cells (Fig. 4 and Figure S4, Supplementary Information). The delivery efficiencies of single proteins ranged between 26% and 71% when cells underwent co-delivery of three types of proteins under the same electroporation procedure. Given that surface charges for all of tested proteins are negative, the large variations in protein delivery efficiency are probably related to protein structures. The aspect ratios of OVA (45 kDa), GFP (27 kDa), and BSA (66 kDa) are 1.4, 1.8 and 3.5, respectively, and the most spherical OVA with a size in between GFP and BSA exhibited highest efficiency. It suggests that intrinsic morphology of the molecules^34^ in addition to molecular weights presumably is a crucial factor to affect the delivery outcome. Their contributions to final delivery efficiency should be carefully examined to enhance overall combinatorial delivery efficiency. Relatively low efficiency for GFP delivery is presumably associated with difficulties in fluorescent signal detections because synthetic Alexa fluorophores may have more stable and stronger fluorescent signals compared to that of GFP. Interestingly, the sequential co-delivery efficiency of dual-proteins ranged from 20% to 31% and that of triple-proteins was 20%. This suggests that the limiting factor for co-delivery efficiency might be protein-type dependent rather than the electroporation parameters. Efficiency enhancement could be achieved if electroporation conditions are optimized for the hardest-to-deliver candidate among all molecules to be co-delivered.

### siRNA and miRNA Delivery

Safety concerns associated with viral-mediated exogenous gene delivery fueled the development of non-viral delivery approaches^35^,^36^,^37^, in particular for those desiring expedited clinical adoptions. RNA interference (RNAi)^38^ exhibits substantial potential for gene therapy and has advanced into clinical stage. siRNAs’ clinical applicability has been demonstrated for the treatment of various cancer, virus infection and genetic disorder^39^. On the other hand, miRNAs have shown to regulate essential genes known to have a key function of cell survival or death and to target multiple oncogenes or pathways simultaneously^13^ with reduced immune response and toxicity^40^. Thus, miRNAs are implicated in the clinical management of diseases, such as cancer^41^,^42^ and Hepatitis C^43^.

To assess μSEA as an alternative gene delivery tool, we first demonstrated gene knockdown using siRNA delivery. MCF7 cells expressing GFP (MCF7-GFP) transfected with GFP-siRNA showed suppression of GFP fluorescent intensity as compared to that of electroporated MCF7-GFP without GFP-siRNA (Figure S5, Supplementary Information). The median fluorescent intensity of the cell population transfected with siRNA decreased to 66% of that of the control (Fig. 5a).

In addition to assessment of gene knockdown, μSEA was utilized for miRNA-mediated phenotype induction by protein regulation using miR-29. The miR-29 family is one of the topmost cancer-associated miRNAs^44^ and miR-29 has been identified as the positive regulators of p53, a tumor suppressor that induces apoptosis by several harmful conditions, through inhibition of p85α and cell division cycle 42 (CDC42)^45^. Control experiments using μSEA revealed that electroporation alone neither affects viability nor alters morphology of processed cells. p53 wild-type cells (MCF7 and HeLa) transfected with hsa-miR-29a-3p (miR-29) exhibited apoptotic phenotype, assessed through colony size measurements and Annexin V staining (Fig. 5b,c, and Figures S6–S7, Supplementary Information). The mean colony size of the entire population for MCF7 and HeLa shrank to 31% and 35% of that of the control, respectively, and their Annexin V intensities were 49% and 58% higher than their control counterparts, respectively. In contrast, no significant difference was found for p53 mutant cell line (MDA-MB-231) for either criterion. Note that cells transfected with miR-29 using μSEA exhibited higher apoptotic tendency although the amount of miRNAs used for μSEA (5 nM of miR-29, 2 pmol per run) was 2-fold lower than that used for lipofectamine-mediated transfection (20 nM of miR-29, 4 pmol per well). The optimum concentration for lipofectamine transfection, 20 nM of miR-29, was determined to be incompatible with μSEA because the transfection induced apoptosis yielded too low cell counts to draw statistically meaningful conclusions at 48 hr post-electroporation. This suggests that μSEA would allow efficient multi-molecular delivery assays with much lower consumption of reagents. The drive to achieve cost-effectiveness is particularly important for assay development^46^ and high throughput screening^47^. Furthermore, cells processed with μSEA are collected in dispersed single-cell manner, implying that the technique would benefit downstream assays preferring single-cell colony formation such as stem cells gene transfection^29^, and the study of gene expression patterns and regulation dynamics at single-cell resolution^30^,^31^.

In summary, we developed a miniaturized electroporation platform for *in vitro* delivery of various biomolecules with high efficiency, viability, and incomparable versatility. μSEA operates at low voltage with negligible cross-chamber voltage variation, enabling the construction of a system with low power consumption. The micropatterned electrodes promote flexibility to integrate with various microfluidic systems while real-time visualization facilitates prompt cell-type dependent fine-tuning of electric parameters, aiming to achieve optimal performance for a wide range of target cells. Various molecules and cell types were utilized to evaluate μSEA’s unique functionalities, including sequential multi-molecular delivery, delivery dosage control and RNA interfering assays. Unlike commercial cuvette-type electroporations, the solution exchange scheme integrated in the μSEA protocol not only eliminates laborious and time-consuming pipetting steps but also removes any post-electroporation by-products that adversely affect cell viability^16^ and downstream analyses. In addition, the system is cost-effective for reagent consumption because transfected cells using μSEA exhibited higher efficiency even though lower amounts of reagents were administered. Moreover, single-cell level transfection allows for the ease of colonogenic assays. In the future, μSEA could purify target cells from complex bodily fluids^26^,^48^, enabling direct administration of molecules into purified target cells, such as circulating tumor cells. μSEA could also be potentially used to process cells with unknown properties since optimum electroporation parameters for such cells could be determined via real-time process visualization. Taken together, we envision μSEA to be a powerful tool for *in vitro* research applications particularly for which combinatorial multi-molecular delivery or circumventing viral-mediated transfection is preferred.

## Methods

### Device Geometry

**Figure 1:**
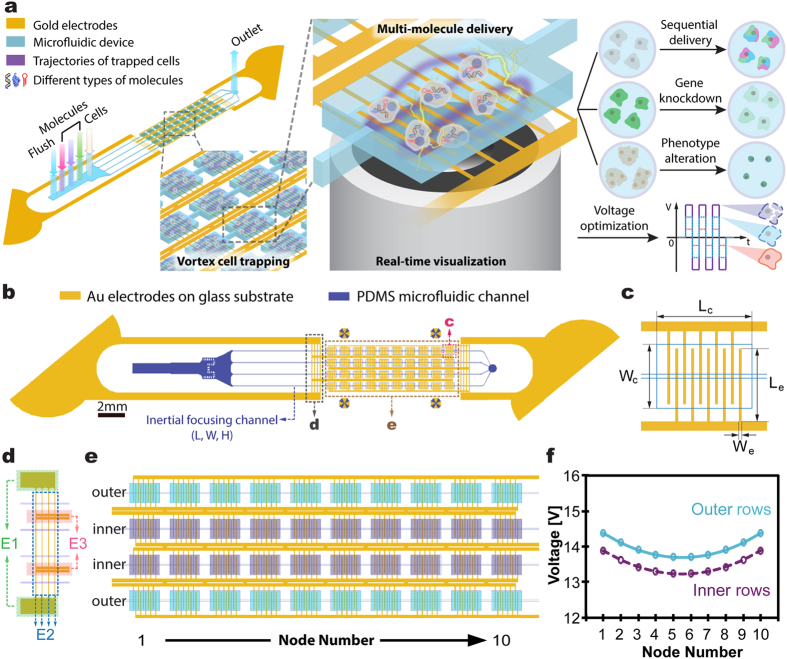
Schematics of μSEA. (**a**) Illustration of the device structure, operational principles and explored applications. (**b**) The assembled microfluidic electroporator, and (**c**) the electrode arrangement within a single cell-trapping chamber. L_c_ = 720 μm; W_c_ = 480 μm; L_e_ = 450 μm; W_e_ = 20 μm. Additional dimensions can be found in Methods in detail. (**d**) The connecting electrode arrangements, and (**e**) the electroporator array. Boxes shaded in purple and turquoise represent cell-trapping microfluidic chambers in inner and outer rows, respectively, while yellow lines indicate Au electrode arrays. (**f**) Cross-chamber voltage variations in outer and inner rows of the chamber array (V_input_ = 20 V) were less than 5%. Each node in this figure represents an electroporation chamber as denoted in (**e**).

**Figure 2:**
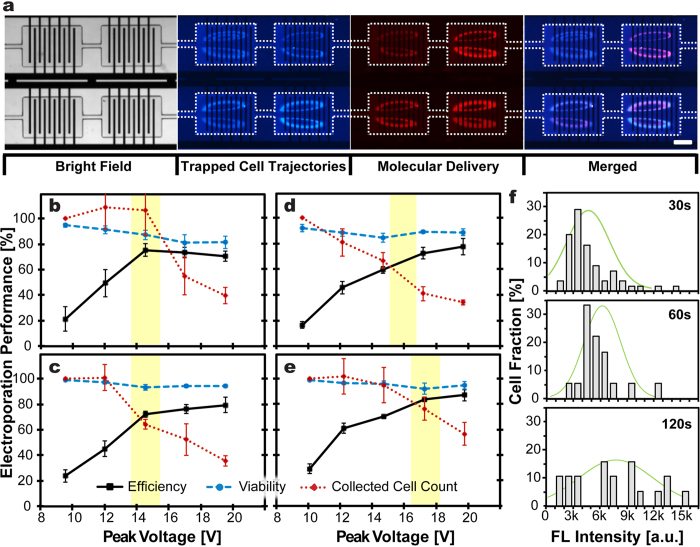
(**a**) Representative microscopic images illustrate successful molecular delivery into trapped cells using μSEA. The white dashed lines outline the cell trapping chambers. HEK 293 cells, pre-stained with Hoechst 33342, were isolated and maintained in the cell trapping orbits in each chamber. Successful delivery of a membrane-impermeable fluorescent molecule Propidium Iodide (PI) into orbiting cells via electroporation was confirmed by detecting fluorescent signals emitted from trapped cells. The results were obtained by electroporating cells with AC square wave pulses with V = 15 V, f = 20 kHz, τ = 1 ms, and Δt = 1 s, followed by 30 s incubation in the PI solution. Scale bar represents 200 μm. (**b**–**e**) Optimization of electroporation parameters for various cell lines. Viability and electroporation efficiency of collected cells for (**b**) HEK293, (**c**) MDA-MB-231, (**d**) HeLa, and (**e**) MCF7 cells. The electroporation parameters that were kept consistent to obtain these results were AC square wave pulses with f = 20 kHz, τ = 1 ms, and Δt = 1 s. Error bars represent standard errors from experiments in triplicates. (n ≥ 280 cells per experiments). (**f**) Histograms of the intensity profile for the HEK293 cells after GFP delivery for delivery dosage control. The delivered amount of GFP increased with increasing incubation durations from 30 s to 120 s. 20 pulses were applied for all three conditions. Green lines overlaid on the histogram represent the Gaussian distribution (n ≥ 18 cells per experiment).

**Figure 3:**
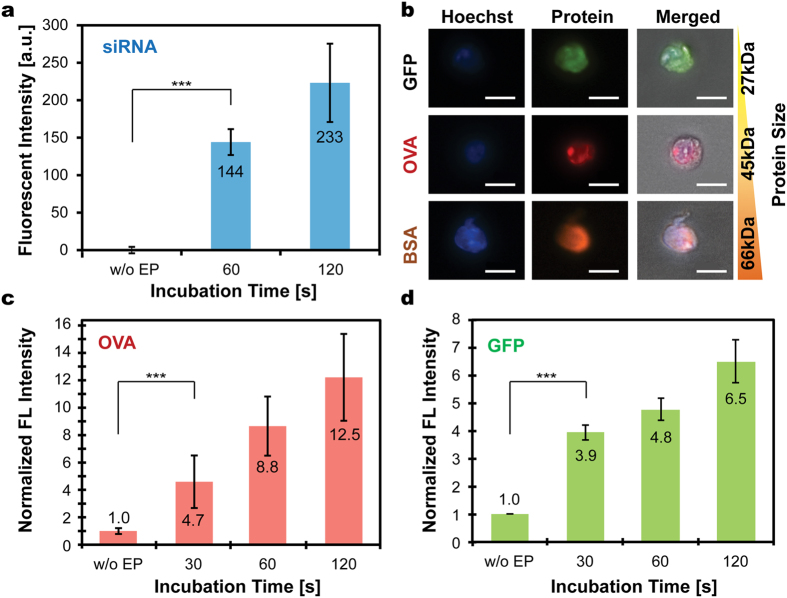
(**a**) Delivered amounts of siRNA conjugated with fluorescein (BLOCK-iT™ Fluorescent Oligo) in HEK293 cells increased with incubation durations and were significantly higher than that of non-electroporated cells (without electroporation, w/o EP). The annotated numbers indicate fluorescent intensity in arbitrary units. (**b**) Microscopic images of processed HEK293 cells indicate successful intracellular delivery of various proteins conjugated with fluorophores. Scale bars represent 20 μm. The tested proteins include green fluorescent protein (GFP), ovalbumin (OVA) conjugated with Alexa 555, and bovine serum albumin (BSA) conjugated with Alexa 647. The delivered amounts of (**c**) OVA and (**d**) GFP in HEK293 cells increased with incubation durations. The annotated numbers indicate fold increases in intensity compared to that of the control. Error bars represent standard errors of electroporated cells (n ≥ 49, 18, 249 cells for (**a**),(**c**) and (**d**), respectively). ***p < 0.001 for control group versus all electroporation conditions, suggesting burst molecular delivery occurs within 30 s, followed by gradual accumulation of delivered amount as incubation duration further increased.

**Figure 4:**
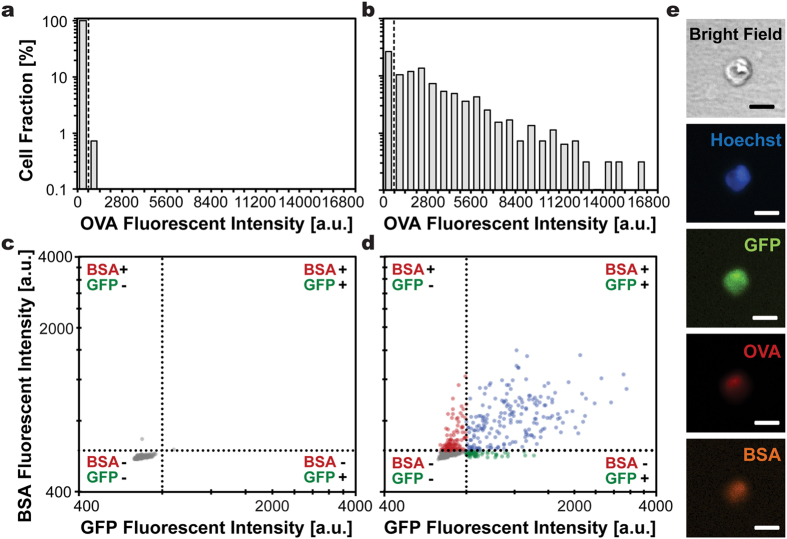
Multiple proteins, including GFP, OVA conjugated with Alexa 555, and BSA conjugated with Alexa 647 were sequentially delivery into HEK293 cells. The histograms illustrate OVA fluorescent intensity exhibited by cells incubated in OVA for 1 min (**a**) without or (**b**) with electroporation. Dashed lines indicate the intensity thresholds above which successful delivery of OVA was determined (Threshold_OVA_ = 700). The cell populations found above the threshold were 0.7% and 73.2% for those processed without or with electroporation, respectively. Cells sequentially incubated in OVA, BSA and GFP (**c**) without electroporation or (**d**) with electroporation exhibited distinctively different fluorescent signals. The fluorescent intensity maps were constructed from (**c**) the same cell population as in (**a**) with 99.3% of the population showing intensities below thresholds, and (**d**) cells with successful OVA delivery (population found above the threshold indicated in (**b**)). The population was divided into 4 subpopulations with circles, respectively, representing cells displaying signals from all three proteins (27%, blue), BSA and OVA (16%, red), GFP and OVA (9%, green), and OVA only (48%, grey). The order of injected molecules was randomized. (n = 272, 963, 705 cells for (**a**,**c**),(**b**) and (**d**), respectively). (**e**) Representative microscopic images illustrate the successful delivery of all three proteins into an identical cell. Scale bars represent 20 μm.

**Figure 5:**
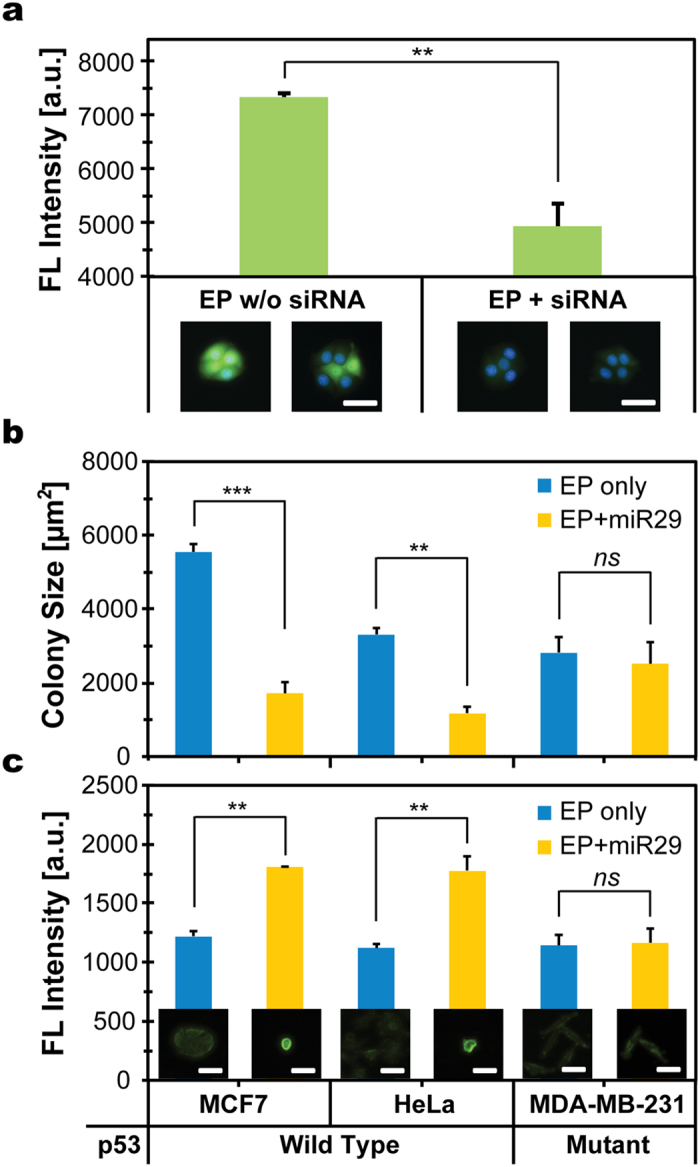
Transfection of RNAi molecules using μSEA revealed that (**a**) GFP-siRNA transfection via electroporation (EP) suppressed GFP expression level of MCF7 cells, and (**b**,**c**) miR-29 transfection selectively induced apoptosis only in cancer cells with wild-type p53. (**a**) Comparison of median fluorescent intensities of single cell colonies from processed cells without or with GFP-siRNA incubation. The representative images of MCF7 cells 2 days post-electroporation are located under the corresponding experimental conditions (n = 598 and 729 cells for without and with siRNA delivery, respectively. (**b**) Colony sizes and (**c**) Annexin V intensities of two p53 wildtype cell lines (MCF7 and HeLa) and a p53 mutant cell line (MDA-MB-231) transfected with miR-29 were compared to those electroporated with a blank solution. Scale bars represent 50 μm. Analyses in (**b**) and (**c**) were obtained from the identical cell population. Error bars represent standard deviation from experiments in triplicates. n = 648 (w/o) and 157; 1217 (w/o) and 337; 1142 (w/o) and 754 cells for MCF7, HeLa, and MDA-MB-231, respectively. *p*-values were determined by two-tailed student *t*-tests. **p < 0.05, ***p < 0.001.

μSEA is composed of two layers: A glass slide with patterned Au electrodes on the surface enclosed with a PDMS layer of cell trapping chamber arrays. Each device includes 40 chambers (4 rows and 10 chambers per row). The microfluidic component of μSEA consists of an inlet with multiple solution injection ports, coarse filters, 4 parallel inertial focusing channels (L = 7 mm, W = 30 μm, and H = 70 μm) and an outlet (Fig. 1b). For all the geometries discussed in this section, the longer dimensions on the device plane indicate the length, whereas shorter ones denote the width. Individual straight inertial focusing channel consists of 10 electroporation chambers in series, which are placed 250 μm apart. There are 5 pairs of interdigitated Au electrodes (W_e_ × L_e_ = 20 μm × 450 μm) underneath each cell-trapping chamber (L_c_ = 720 μm; W_c_ = 480 μm; H_c_ = 70 μm) (Fig. 1c). For each row of the cell trapping chambers, electrodes with the same polarity are connected to a single wire transferring electric signals from the source (denoted as *E3* in Fig. 1d, W_E3_ = 80 μm). The connecting wires were designed to have two sections of varied electrode widths. The length and width of the first electrode section (denoted as *E1* in Fig. 1d) are L_E1_ ≈ 16 mm and W_E1_ = 500 μm, respectively. These electrodes are then branched into the second set of 4 connecting electrodes (denoted as *E2* in Fig. 1d) with length, L_E2_ ≈ 3 mm, and width, W_E2_ = 20 μm. The width of 20 μm is critical to irreversibly seal the PDMS device onto a glass slide with micropatterned 300 nm-thick Au electrodes, ensuring no leakage under high operational pressure (>30 psi). The purpose of the parallel configuration of *E2* (4 × 20 μm), instead of a single wide electrode design (1 × 80 μm), was to eliminate leakage at the contact surface between Au electrodes and the PDMS layer without increasing the overall electrical resistance across these microscale electrodes.

### Device Fabrication

Two-dimensional projections of the microfluidic chambers and the electrode geometry were designed using AutoCAD (Autodesk, Inc., USA) and the CAD file was converted to a GDSII file using LinkCAD. The micro-patterns were written on a 5 in × 5 in photomask blank using a laser mask writer (Heidelberg Mask Writer, DWL-66). The mask was developed following the manufacturer’s protocol. A negative photoresist (KMPR 1050, Microchem, USA) was used to fabricate the casting mold by following the standard soft lithography technique. The heights of fabricated microstructures were measured using a surface profiler (Dektak 6 M, Veeco, USA). Polydimethylsiloxane (PDMS, Sylgard^®^ 184 silicone elastomer kit, Dow corning, USA) replicas were created by mixing base and curing agents at 5:1 ratio. The mixture was degassed for 45 min and cured at room temperature on a leveled surface for 24 hr. We deliberately chose to cure PDMS at room temperature to avoid misalignment induced by geometry changes due to escalated thermal shrinkage at higher temperature. The solution injection ports and outlet were created using a pin vise (Pin vise set A with a 20 G punch, Syneo, LLC.). The Au electrode array was fabricated by a lift-off process using a positive photoresist (S1813, Microchem, USA). The electrodes were created by depositing 10 nm Cr and 300 nm Au on glass slides using an e-beam evaporator (Denton, USA). The microchannels were enclosed by bonding PDMS replicas to glass slides with patterned Au electrodes using oxygen-plasma treatment (Technics Micro-RIE, USA) at 75 W, 500 mTorr for 7 s.

### Experimental Setup

The experimental setup for electroporation consists of (i) an inverted microscope (Eclipse Ti, Nikon Inc., Japan) for real-time visualization of electroporation processes, (ii) the in-house built pneumatic flow control unit for pressurizing individual solution vials, enabling rapid solution exchanges through the microfluidic system, and (iii) electronic components to apply AC square wave. The microscope is equipped with an Epi-fluorescent illuminator (Intensilight, Nikon Inc., Japan), fluorescent filter cube sets, and a CCD camera (Clara, Andor, USA). The electronic components include a pulse generator (HP 8110 A), a function generator (HP 33120 A), a source meter (HP E3630A), a custom-designed square-wave generator, and an oscilloscope (Agilent, USA). More details of the pneumatic flow control unit and system setup have been previously reported and can be found elsewhere^24^.

### Electroporation Procedure

Multiple vials individually containing cells and reagents were mounted to the custom-built pneumatic flow control unit. PEEK tubings connected to each solution vial were inserted into the designated solution injection ports on the device for individual flow control. Additional PEEK tubing was connected to the outlet port on the device for downstream sample collections. The device was firstly flushed with Dulbecco’s Phosphate Buffered Saline Solution (DPBS, HyClone™ GE Healthcare Life Sciences, USA) at 30 psi for 30 s to initiate and stabilize vortex-formations. Then, cell solution was injected into the microchannel at the operational pressure of 35 psi (equivalent to a flow rate of 1.6 mL/min for the 4-parallel-channel device) for 60 s for efficient cell trapping. After the cell number trapped inside the micro-vortices was equilibrated, the active solution port was switched from the cell solution to the flush to remove cells that are not stably trapped in vortices. The solution containing a substance to be delivered was then flown into the device at 35 psi. 10 s post molecular injection, the electroporation process was initiated by applying desired electrical pulses to the trapped cells while they are incubated in the molecule of interest. All voltage, V, listed in this manuscript represents peak voltage, V_pk_. A pulse is defined as a burst of 20 cycles at 20 kHz lasting for 1 ms. Unless specified otherwise, typical parameters utilized for electroporation are AC square waves with V_pk_ ranging from 12 to 20 V, frequency, f = 20 kHz, pulse width, τ = 1 ms, and pulse interval, Δt = 1 s. An oscilloscope (Agilent, USA) was employed to monitor pulse waveforms and to measure the magnitudes of voltages, V_pk_, as well as currents, I, applied across the device in real-time. Upon completion of the electroporation process, the microfluidic system was flushed with DPBS at 35 psi for 30 s to remove residual reagent while maintaining the electroporated cell in vortices. The treated cells were then released from the trapping chambers by lowering the operating pressure to 15 psi and subsequently collected in a 96 well-plate.

### Cell Preparation

HeLa, MCF7, HEK293, and MDA-MB-231 cell lines were purchased from ATCC. MCF7 expressing GFP (MCF7-GFP) was kindly provided by Prof. Stefanie Jeffrey’s Lab at Stanford University. Cells were plated with 10 mL of growth media in T75 flasks (Corning Inc., USA) at a concentration of 1 × 10^5^ cells/mL. The growth media for HeLa, HEK293, MCF7 and MCF7-GFP was composed of Dulbecco’s Modified Eagle Medium (DMEM, Gibco^®^, Life technologies, USA) supplemented with 10% (v/v) fetal bovine serum (FBS, Gibco^®^, Life technologies, USA) and 1% penicillin-streptomycin (Sigma-Aldrich Co., USA). Cells were grown in a humidified incubator at 37 °C with 5% CO_2_ environment. Metastatic breast cancer cells, MDA-MB-231, were maintained in Leibovitz’s L-15 Medium (Cellgro^®^, Mediatech, Inc., USA) supplemented with 10% (v/v) FBS and 1% penicillin-streptomycin (Sigma-Aldrich Co., USA). MDA-MB-231 cell line was incubated in a humidified incubator at 37 °C with 0% CO_2_ environment. Cells were harvested by treating with 0.25% trypsin-EDTA (Gibco^®^, Life technologies, USA) for 3 min 1–2 days after seeding. Then, cells were pelleted by centrifuging for 5 min at 1200 rpm and resuspended into the growth media to have a final concentration of 5 × 10^5^ cells/mL for electroporation.

### Optimization of Electroporation Parameters

Cells were pre-stained with Hoechst 33342 (NucBlue^®^, Thermo Fisher Scientific Inc., USA) prior to electroporation to visualize cellular trajectories upon successful trapping. A membrane-impermeable fluorescent molecule Propidium Iodide (PI, Life technologies, USA) was used to evaluate electroporation efficiency. The electroporation parameter was 10 pulses followed by additional 30 s incubation in 15 μM PI solution. Electroporated cells were collected downstream into 96 well plates and stained with 1.1 μM Calcium Green AM (Life technologies, USA) to evaluate their immediate post-electroporation viability (<1 hr). Collected cell count is defined by counting all collected cells, exhibiting Hoechst 33342 signals. Viability is defined as the percentage of cells, exhibiting Calcein Green AM signal among collected cells. Efficiency represents the percentage of cells, exhibiting both PI and Calcein Green acetoxymethyl (AM) signals among collected cells. Only in viable cells, the non-fluorescent Calcein AM is converted into membrane-impermeable green-fluorescent Calcein molecules via hydrolysis of esterified subgroups of acetoxymethyl, providing a means to visually evaluate membrane integrity and viability post-electroporation. Calcein AM/PI immunofluorescent staining and dead cell exclusion methods were performed for rapid and single cell level viability assessments because other conventional assays such as MTT assay utilize absorbance variations originated from ensemble of cells in the well^49^ and individual cells electroporated using μSEA may generate too subtle absorbance variations to be accurately and quantitatively distinguished among different experimental groups.

### Protein and Protein-Conjugate Delivery

Renilla Reniformis GFP was purchased from NanoLight^®^ Technologies, Prolume Ltd., USA. Ovalbumin (OVA) conjugated with Alexa 555 (O34782) and bovine serum albumin (BSA) conjugated with Alexa 647 (A34785) were purchased from Invitrogen^TM^, Thermo Fisher Scientific Inc., USA. The electroporation parameter used for protein delivery into HEK293 cell line was 20 pulses at 15 V followed by additional 20 s incubation in 600 nM protein conjugate solutions (i.e., 1 min incubation in total). The microfluidic control experiment to determine the baseline intensity threshold was performed by incubating the trapped cells for 1 min in the protein conjugate solution with a concentration identical to those used for electroporation.

### siRNA Delivery

For well plate control, Lipofectamine^®^ LTX with Plus™ Reagent (Invitrogen^TM^, Thermo Fisher Scientific Inc., USA) was used to transfect MCF7-GFP with Silencer^®^ GFP siRNA (siRNA, Ambion™, Thermo Fisher Scientific Inc., USA) one day after seeding (initial seeding concentration of 5 k cells/well in the 96 well plate) by following the manufacturer’s protocol. siRNA concentrations ranging from 40 nM to 100 nM were tested to determine Lipofectamine transfection efficiency. 80 nM was chosen as the concentration to compare efficiencies of lipofectamine- and electroporation-mediated transfections because noticeable GFP expression knockdown was observed for siRNA concentrations above 80 nM for lipofectamine transfections. For electroporation, MCF7-GFP cells were exposed to 20 pulses at 17 V, followed by 20 s incubation in the 80 nM siRNA solution. The total 1 min incubation utilizes 32 pmol of siRNA per microchannel. Electroporated cells were collected in a 96 well plate immediately post-electroporation. The microfluidic control experiment was performed by incubating the cells in the 80 nM siRNA solution for 1 min without electroporation. 48 hr after transfection, cells were washed with DPBS and stained with NucBlue^®^ for viability assessment. GFP expression levels of treated cells were further accessed via quantitative analyses of fluorescent microscopic images.

### miRNA Delivery

p53 wild-type (MCF7 and HeLa) and mutant (MDA-MB-231) cells were transfected with hsa-miR-29a-3p (miR-29, HMI0434, Sigma-Aldrich^®^) via Lipofectamine^®^ LTX with Plus™ Reagent as the control experiment. 20 nM and 5 nM of miR-29 were used for lipofectamine- and electroporation-mediated transfections, respectively. The electroporation procedure and parameters were similar to that of siRNA delivery. The optimal voltages (17, 16, and 15 V for MCF7, HeLa and MDA-MB-231, respectively) were used for each cell line. The microfluidic control experiment was performed by incubating the cells in a 5 nM miR-29 solution for 1 min without electroporation. 48 hr after transfection, cells in the well plate were washed with DPBS and fixed with 4% (v/v) paraformaldehyde for 5 min at room temperature. Then, cells were washed twice with DPBS and stained with fluorescein isothioyanate (FITC) – conjugated Annexin V (Molecular Probes™, Thermo Fisher Scientific Inc., USA) diluted in a binding buffer (Molecular Probes™, Thermo Fisher Scientific Inc., USA) for 1 hour by following the manufacturer’s protocol. Stained cells were visualized by fluorescence microscopy to quantify cells exhibiting apoptotic phenotypes. In addition to Annexin V staining, we evaluated transfected cells’ apoptotic volume decrease (AVD), which is one of the ubiquitous aspects for apoptotic phenotypes depicted by the loss of cell volume or cell shrinkage^50^, by measuring the colony size as an indication of the 2D projection of AVD.

### Quantitative Analyses of Fluorescent Images

Fluorescent microscopic images of the collected cells were captured using 10× objective by setting up multi-point automated sequences to image the entire well in 96 well plates. In order to quantitatively evaluate fluorescent intensities or colony sizes, the region of interest (ROI) was defined by drawing outlines around cells of interest and individual ROIs were analyzed using NIS Elements software (Nikon Inc., Japan). The thresholds for successful protein delivery were determined for each protein type respectively such that intensities emitted by more than 99% of the cells in the control group were found below the threshold. These threshold values were defined to be greater than *I*_*mean*_ + *3σ*, where *I*_*mean*_ and *σ* are the average intensity and the standard deviation of the entire processed cell populations in the control group, respectively. For siRNA and miR-29 delivery experiments, histogram of fluorescent signals from the entire cell population was analyzed, thus no fluorescent threshold was defined.

### Pulse Driver Mechanism

We built a custom bipolar pulse driver for μSEA, which accepts a programmed low-voltage bipolar signal from an Agilent 33120A Arbitrary Waveform Generator, and creates high-power voltage pulses (Figure S8, Supplementary Information). It can deliver up to 2 A to μSEA, with the capability to support up to 160 chambers (4 times of the current configuration). The bipolar voltages + V and –V are adjusted up to ± 30 V with an Agilent E3630A Tracking Power Supply. Transistors Q1 or Q2 create a 40 mA level-shifting current, and in turn a 6 V gate drives across a 150 Ω resistor^51^. This is applied through a diode to turn on MOSFET Q5 or Q6. At the end of each pulse half-cycle, transistors Q3 or Q4 rapidly discharge the high gate capacitance (1.5 nF) and turn off the MOSFET. The Waveform Generator is programmed to have a short deadtime between polarity changes.

## Additional Information

**How to cite this article:** Ouyang, M. *et al*. Microscale Symmetrical Electroporator Array as a Versatile Molecular Delivery System. *Sci. Rep.* **7**, 44757; doi: 10.1038/srep44757 (2017).

**Publisher's note:** Springer Nature remains neutral with regard to jurisdictional claims in published maps and institutional affiliations.

## Supplementary Material

Supplementary Information

## References

[b1] Morales D. P. . Targeted Intracellular Delivery of Proteins with Spatial and Temporal Control. Mol Pharmaceut 12, 600–609 (2015). 10.1021/mp500675p PMC4319691 25490248

[b2] Yan M. . A novel intracellular protein delivery platform based on single-protein nanocapsules. Nat Nanotechnol 5, 48–53 (2010). 19935648 10.1038/nnano.2009.341

[b3] Ibraheem D., Elaissari A. & Fessi H. Gene therapy and DNA delivery systems. International journal of pharmaceutics 459, 70–83 (2014). 24286924 10.1016/j.ijpharm.2013.11.041

[b4] Luo D. & Saltzman W. M. Synthetic DNA delivery systems. Nat Biotechnol 18, 33–37 (2000). 10625387 10.1038/71889

[b5] Lee J. B. . Self-assembled RNA interference microsponges for efficient siRNA delivery. Nature materials 11, 316–322 (2012). 22367004 10.1038/nmat3253 PMC3965374

[b6] Whitehead K. A., Langer R. & Anderson D. G. Knocking down barriers: advances in siRNA delivery. Nature reviews. Drug discovery 8, 129–138 (2009). 19180106 10.1038/nrd2742 PMC7097568

[b7] Mendez-Ortega M. C. . An RNAi in silico approach to find an optimal shRNA cocktail against HIV-1. Virol J 7, 369 (2010). 21172023 10.1186/1743-422X-7-369 PMC3022682

[b8] Meng J. R. . Combination Treatment with MEK and AKT Inhibitors Is More Effective than Each Drug Alone in Human Non-Small Cell Lung Cancer *In vitro* and *In vivo*. Plos One 5, e14124 (2010). 21124782 10.1371/journal.pone.0014124 PMC2993951

[b9] Wu Z. K., Zhao X. M. & Chen L. N. A systems biology approach to identify effective cocktail drugs. Bmc Syst Biol 4 (Suppl 2), S7 (2010). 10.1186/1752-0509-4-S2-S7 PMC2982694 20840734

[b10] Takahashi K. & Yamanaka S. Induction of pluripotent stem cells from mouse embryonic and adult fibroblast cultures by defined factors. Cell 126, 663–676 (2006). 16904174 10.1016/j.cell.2006.07.024

[b11] Feng B., Ng J. H., Heng J. C. D. & Ng H. H. Molecules that Promote or Enhance Reprogramming of Somatic Cells to Induced Pluripotent Stem Cells. Cell Stem Cell 4, 301–312 (2009). 19341620 10.1016/j.stem.2009.03.005

[b12] Soldner F. . Parkinson’s Disease Patient-Derived Induced Pluripotent Stem Cells Free of Viral Reprogramming Factors (vol 136, pg 964, 2009). Cell 137, 1356–1356 (2009). 10.1016/j.cell.2009.02.013 PMC2787236 19269371

[b13] Pereira D. M., Rodrigues P. M., Borralho P. M. & Rodrigues C. M. P. Delivering the promise of miRNA cancer therapeutics. Drug Discov Today 18, 282–289 (2013). 23064097 10.1016/j.drudis.2012.10.002

[b14] Saraswathy M. & Gong S. Q. Recent developments in the co-delivery of siRNA and small molecule anticancer drugs for cancer treatment. Mater Today 17, 298–306 (2014).

[b15] Weaver J. C. & Chizmadzhev Y. A. Theory of electroporation: A review. Bioelectroch Bioener 41, 135–160 (1996).

[b16] Costa M. . A method for genetic modification of human embryonic stem cells using electroporation. Nat Protoc 2, 792–796 (2007). 17446878 10.1038/nprot.2007.105

[b17] Chen G. K. . Chemically defined conditions for human iPSC derivation and culture. Nat Methods 8, 424–U476 (2011). 21478862 10.1038/nmeth.1593 PMC3084903

[b18] Lee W. G., Demirci U. & Khademhosseini A. Microscale electroporation: challenges and perspectives for clinical applications. Integr Biol 1, 242–251 (2009). 10.1039/b819201d PMC3771519 20023735

[b19] Movahed S. & Li D. Q. Microfluidics cell electroporation. Microfluid Nanofluid 10, 703–734 (2011).

[b20] Geng T. & Lu C. Microfluidic electroporation for cellular analysis and delivery. Lab Chip 13, 3803–3821 (2013). 23917998 10.1039/c3lc50566a PMC3787074

[b21] Yang Z. G., Chang L. Q., Chiang C. L. & Lee L. J. Micro-/Nano-Electroporation for Active Gene Delivery. Curr Pharm Design 21, 6081–6088 (2015). 10.2174/1381612821666151027152121 26503150

[b22] Kotnik T. . Electroporation-based applications in biotechnology. Trends Biotechnol 33, 480–488 (2015). 26116227 10.1016/j.tibtech.2015.06.002

[b23] Zheng M. D. . Hydrodynamically controlled cell rotation in an electroporation microchip to circumferentially deliver molecules into single cells. Microfluid Nanofluid 20 (2016).

[b24] Yun H. Y. & Hur S. C. Sequential multi-molecule delivery using vortex-assisted electroporation. Lab Chip 13, 2764–2772 (2013). 23727978 10.1039/c3lc50196e

[b25] Vickers D. A. L., Ouyang M. X., Choi C. H. & Hur S. C. Direct Drug Cocktail Analyses Using Microscale Vortex-Assisted Electroporation. Anal Chem 86, 10099–10105 (2014). 25291206 10.1021/ac501479g

[b26] Sollier E. . Size-selective collection of circulating tumor cells using Vortex technology. Lab Chip 14, 63–77 (2014). 24061411 10.1039/c3lc50689d

[b27] Lu H., Schmidt M. A. & Jensen K. F. A microfluidic electroporation device for cell lysis. Lab Chip 5, 23–29 (2005). 15616736 10.1039/b406205a

[b28] Ziv R. . Micro-electroporation of mesenchymal stem cells with alternating electrical current pulses. Biomedical microdevices 11, 95–101 (2009). 18815886 10.1007/s10544-008-9213-4

[b29] Nii T. . Single-Cell-State Culture of Human Pluripotent Stem Cells Increases Transfection Efficiency. BioResearch open access 5, 127–136 (2016). 27257519 10.1089/biores.2016.0009 PMC4876534

[b30] Kim T. K. & Eberwine J. H. Mammalian cell transfection: the present and the future. Analytical and bioanalytical chemistry 397, 3173–3178 (2010). 20549496 10.1007/s00216-010-3821-6 PMC2911531

[b31] Bennett M. R. & Hasty J. Microfluidic devices for measuring gene network dynamics in single cells. Nature reviews. Genetics 10, 628–638 (2009). 10.1038/nrg2625 PMC2931582 19668248

[b32] Mura S., Nicolas J. & Couvreur P. Stimuli-responsive nanocarriers for drug delivery. Nature materials 12, 991–1003 (2013). 24150417 10.1038/nmat3776

[b33] Wang S. & Lee L. J. Micro-/nanofluidics based cell electroporation. Biomicrofluidics 7, 11301 (2013). 23405056 10.1063/1.4774071 PMC3555966

[b34] Chithrani B. D. & Chan W. C. W. Elucidating the mechanism of cellular uptake and removal of protein-coated gold nanoparticles of different sizes and shapes. Nano Lett 7, 1542–1550 (2007). 17465586 10.1021/nl070363y

[b35] Wu Y. C. . Massively parallel delivery of large cargo into mammalian cells with light pulses. Nat Methods 12, 439–444 (2015). 25849636 10.1038/nmeth.3357 PMC5082232

[b36] Boukany P. E. . Nanochannel electroporation delivers precise amounts of biomolecules into living cells. Nat Nanotechnol 6, 747–754 (2011). 22002097 10.1038/nnano.2011.164

[b37] Sharei A. . A vector-free microfluidic platform for intracellular delivery. P Natl Acad Sci USA 110, 2082–2087 (2013). 10.1073/pnas.1218705110 PMC3568376 23341631

[b38] Novina C. D. & Sharp P. A. The RNAi revolution. Nature 430, 161–164 (2004). 15241403 10.1038/430161a

[b39] Lam J. K. W., Chow M. Y. T., Zhang Y. & Leung, S. W. S. siRNA Versus miRNA as Therapeutics for Gene Silencing. Mol Ther-Nucl Acids 4, e252 (2015). 10.1038/mtna.2015.23 PMC4877448 26372022

[b40] Chen Y. C., Gao D. Y. & Huang L. *In vivo* delivery of miRNAs for cancer therapy: Challenges and strategies. Adv Drug Deliver Rev 81, 128–141 (2015). 10.1016/j.addr.2014.05.009 PMC5009470 24859533

[b41] Hayes J., Peruzzi P. P. & Lawler S. MicroRNAs in cancer: biomarkers, functions and therapy. Trends Mol Med 20, 460–469 (2014). 25027972 10.1016/j.molmed.2014.06.005

[b42] Hansen T. F. . MicroRNA-126 and epidermal growth factor-like domain 7-an angiogenic couple of importance in metastatic colorectal cancer. Results from the Nordic ACT trial. Br J Cancer 109, 1243–1251 (2013). 23922111 10.1038/bjc.2013.448 PMC3778299

[b43] Janssen H. L. . Treatment of HCV infection by targeting microRNA. The New England journal of medicine 368, 1685–1694 (2013). 23534542 10.1056/NEJMoa1209026

[b44] Spizzo R., Nicoloso M. S., Croce C. M. & Calin G. A. SnapShot: MicroRNAs in Cancer. Cell 137, 586–U204 (2009). 19410551 10.1016/j.cell.2009.04.040

[b45] Park S. Y. . miR-29 miRNAs activate p53 by targeting p85 alpha and CDC42. Nature structural & molecular biology 16, 23–29 (2009). 10.1038/nsmb.1533 19079265

[b46] Baker M. Academic screening goes high-throughput. Nat Methods 7, 787–792 (2010).

[b47] Macarron R. . Impact of high-throughput screening in biomedical research. Nature reviews. Drug discovery 10, 188–195 (2011). 21358738 10.1038/nrd3368

[b48] Che J. . Classification of large circulating tumor cells isolated with ultra-high throughput microfluidic Vortex technology. Oncotarget 7, 12748–12760 (2016). 26863573 10.18632/oncotarget.7220 PMC4914319

[b49] Halter M. Modernizing the MTT assay with microfluidic technology and image cytometry. Cytom Part A 81A, 643–645 (2012). 10.1002/cyto.a.22089 22730082

[b50] Bortner C. D. & Cidlowski J. A. Apoptotic volume decrease and the incredible shrinking cell. Cell Death Differ 9, 1307–1310 (2002). 12478467 10.1038/sj.cdd.4401126

[b51] Horowitz P. & Hill W. The Art of Electronics, 3rd Edition. 193, 204 (Cambridge University Press, 2015).

