# Supplementary material for: Microscale Symmetrical Electroporator Array as a Versatile Molecular Delivery System
Source: Sci Rep. 2017 Mar 20;7:44757. doi: 10.1038/srep44757 (PMC5357946; doi:10.1038/srep44757)
Supplement: Supplementary Information [file srep44757-s1.pdf]

Supplementary Information

Microscale Symmetrical Electroporator Array as a Versatile Molecular Delivery System

Mengxing Ouyang, Winfield Hill, Jung Hyun Lee, and Soojung Claire Hur*

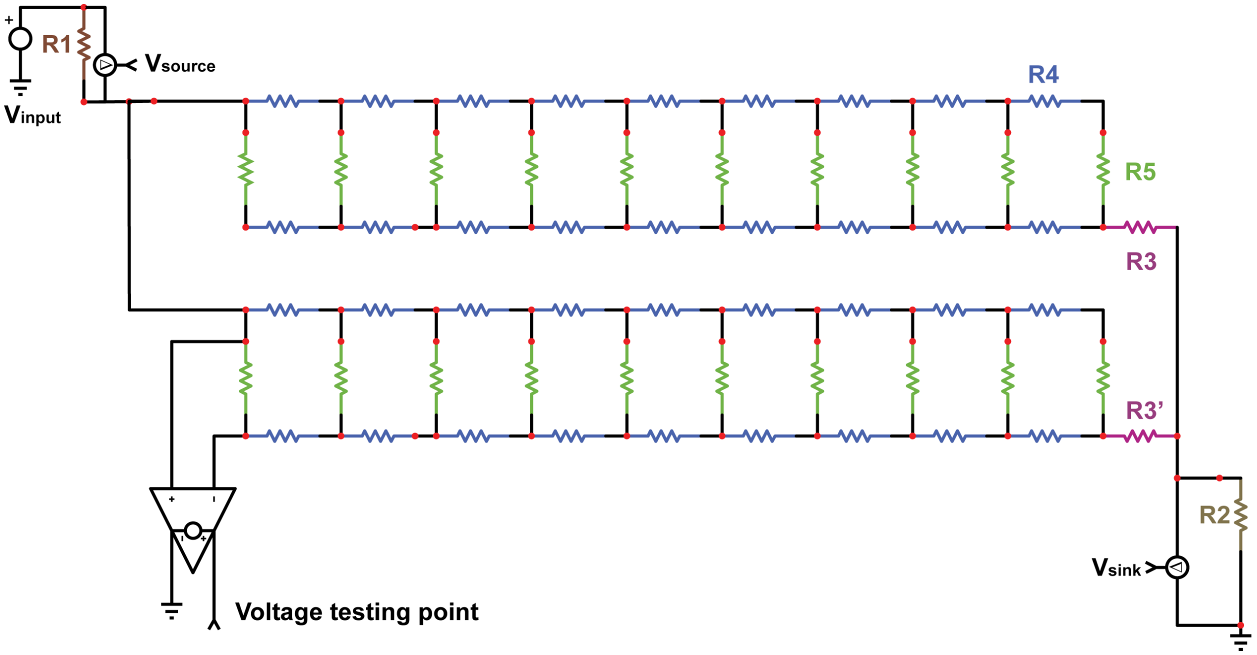

Supplementary Figure S1. The SPICE model of the upper half of the electrode array. The other half of the device shares the identical structures. R1 represents the overall resistance of the shared connecting electrodes between the inlet-side electrode pad and electroporator array along electrodes at the location of *E1*, *E2* and *E3* as denoted in **Fig. 1d**. R2 represents the overall resistance of the shared connecting electrodes between electroporator array and the outlet-side electrode pad along *E1*. R3 and R3' represent the resistance of the residual connecting electrodes along *E2* and *E3* for outer and inner row, respectively. R4 represents the resistance of the electrodes on the common bus line between adjacent chambers. R5 represents the resistance of the trapping unit including individual trapping chamber and the extension of 20 μm electrodes. The resistance of the Au electrodes is calculated as $R = \rho l/A$, where ρ, l, and A represent the resistivity of Au (2.20×10^{-8} Ω·m), the length of the electrode, and the cross-sectional area of the electrode, respectively. The values estimated by the SPICE model based on electrode geometry were R1 = 4.306 Ω, R2 = 1.011 Ω, R3 = 1.267 Ω, R3' = 2.919 Ω, R4 = 0.986 Ω, and R5 = 401.8 Ω. The electrical resistance of the individual trapping chamber filled with Dulbecco's phosphate-buffered saline (DPBS) is estimated to be 401.6 Ω from COMSOL simulation **(Supplementary Fig. 2)**. Voltage testing points were added to each node to measure the value of voltage drop across the nodes as required. The overall resistance of each row of the electroporator array (10 chambers) was measured to be 45.8 Ω.

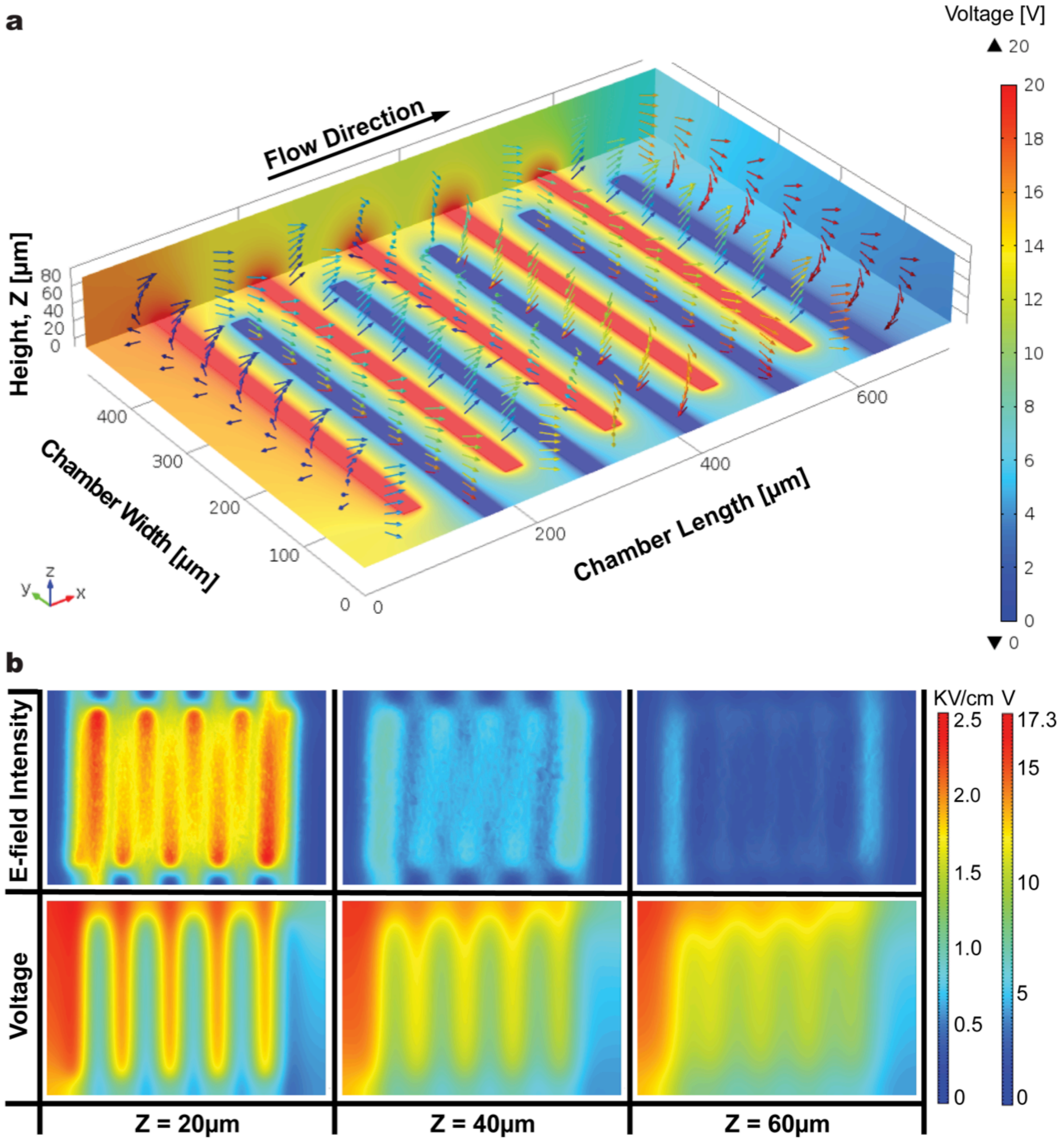

Supplementary Figure S2. COMSOL simulations were conducted to estimate **(a)** electric potential, denoted by surface colors, and electric field distribution, denoted by colored arrows, in a single chamber, and **(b)** electric potential and electric field intensity distribution at different heights away from the electrode surfaces. Prior to series of empirical investigations, the simulation is conducted to estimate a minimum operational voltage to achieve sufficient electric field intensity for successful electroporation at Z = 60 µm which is the furthest distance from the electrodes, potentially occupied by orbiting cells. The simulation was conducted with the Electric Currents mode using COMSOL Multiphysics® software (version 4.4). The fluid is assumed to have the properties of DPBS at room temperature and the values used for the solution's density, dynamic viscosity, and electrical conductivity were 1000 kg/m^3, 1.005 mPa·s, and 1.4 S/m, respectively. The density and electrical conductivity of Au electrodes were set as 19, 300 kg/m^3, and 4.56 × 10^7 S/m, respectively. The applied voltage was set as 20 V. The model predicted the resistance of the single chamber filled with DPBS to be 401.6 Ω.

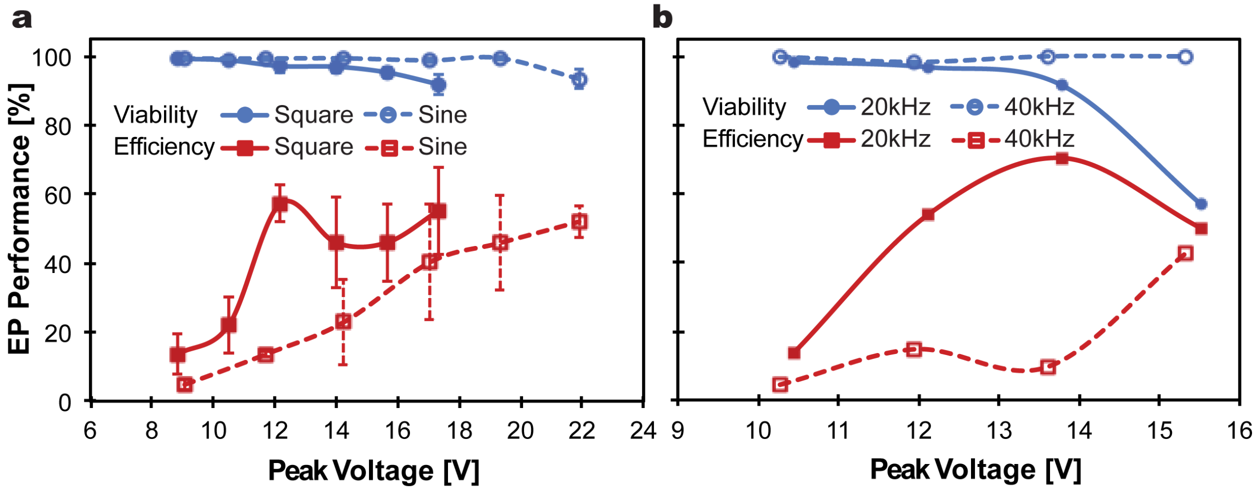

Supplementary Figure S3. The cell viability and electroporation efficiency of HEK293 cells were evaluated in terms of **(a)** sine wave vs. square wave at 20 kHz and **(b)** square waves at 20 kHz vs. 40 kHz. Square waveform was chosen over sine waveform because (i) it exhibited slightly better electroporation efficiency when the identical peak AC voltage was applied, and (ii) it would provide simplicity in voltage source electronic circuitry. Constant parameters were $\tau = 1$ ms and $\Delta t = 1$ s. Error bars in (b) represent standard errors from experiments in triplicate.

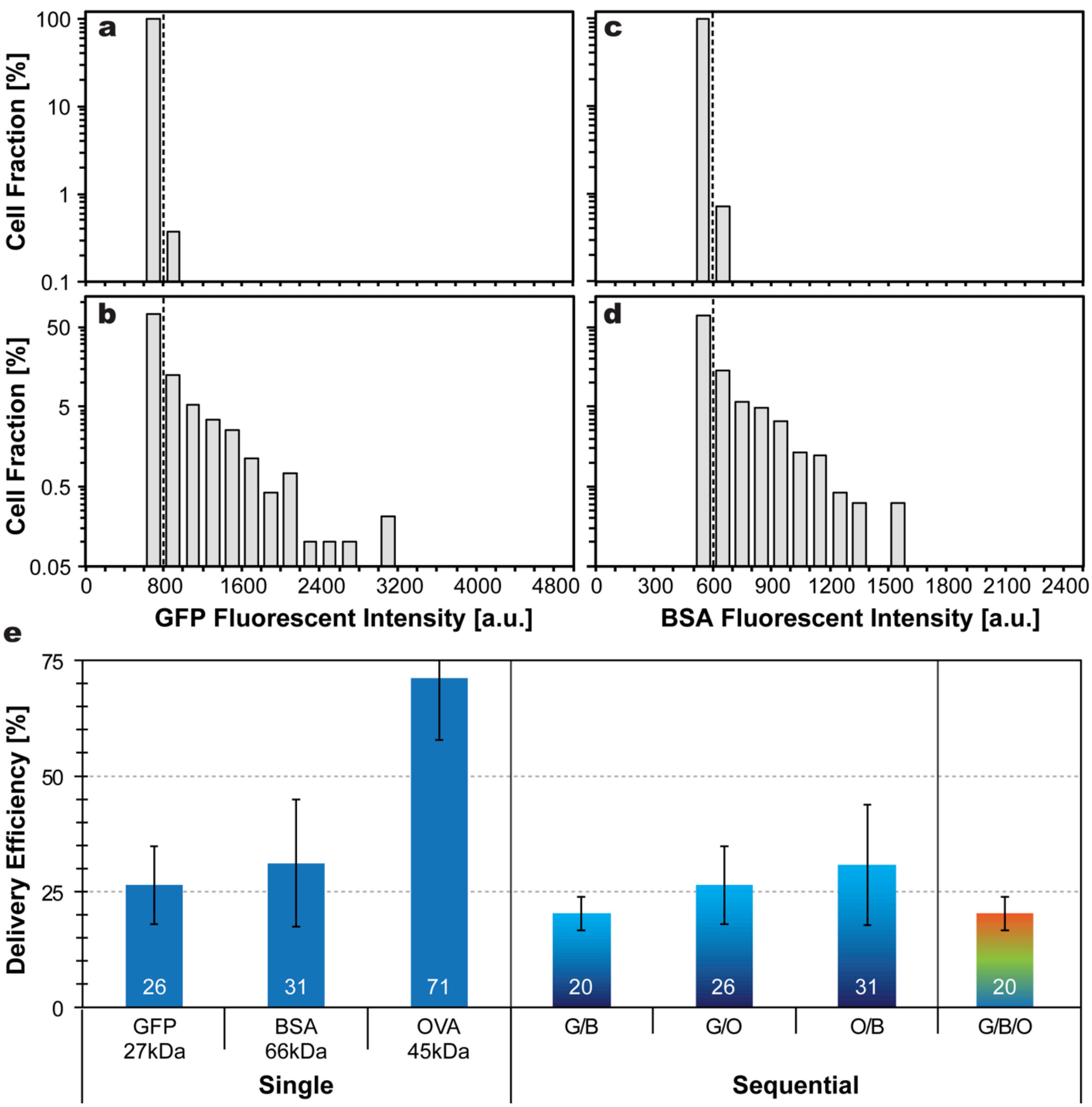

Supplementary Figure S4. Sequential co-delivery of GFP, OVA-Alexa 555, and BSA-Alexa 647 into HEK293. The fluorescent intensity histograms of cells incubated in GFP **(a)** without or **(b)** with electroporation. The fluorescent intensity histograms of cells incubated in BSA-Alexa 647 **(c)** without or **(d)** with electroporation. Dashed lines indicate the intensity threshold for successful protein delivery, below which 99.6% and 99.3% of the cell population incubated in GFP and OVA without electroporation were found, respectively. The intensity threshold values for GFP and BSA were 800 and 600, respectively. **(e)** Among all cell populations underwent the co-delivery of three proteins, the efficiencies of single, dual and triple protein delivery were evaluated and compared for HEK293 cells. Error bars represent standard deviation from experiments in triplicate. n = 272, 963 cells for (a, c) and (b, d, e), respectively. The co-delivery efficiency is bounded by the lowest of delivered proteins regardless of the number of delivered proteins, hence, can be increased by improving the delivery of the protein with the lowest efficiency. The order of injected molecules was randomized.

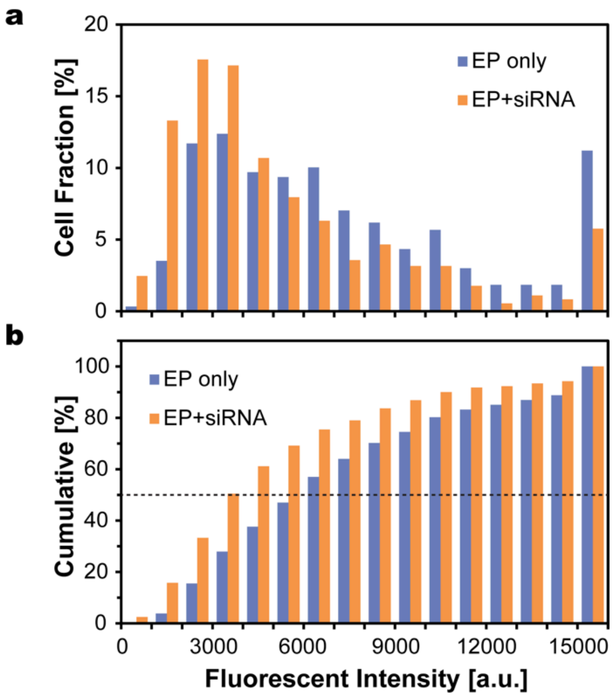

Supplementary Figure S5. GFP-siRNA transfection via electroporation (EP) suppressed GFP expression level of MCF7-GFP cells. **(a)** The fluorescent intensity histogram and **(b)** cumulative cell fraction histogram obtained for cells electroporated without or with the presence of GFP-siRNA. The shift of overall histogram towards lower fluorescent intensity suggests that the successful GFP-siRNA transfection reduced the GFP expression. In the cumulative histogram, the fluorescent intensities at 50% cell population for both groups were identified and compared in **Fig. 3a**. n = 598 and 729 cells for electroporation without or with siRNA, respectively.

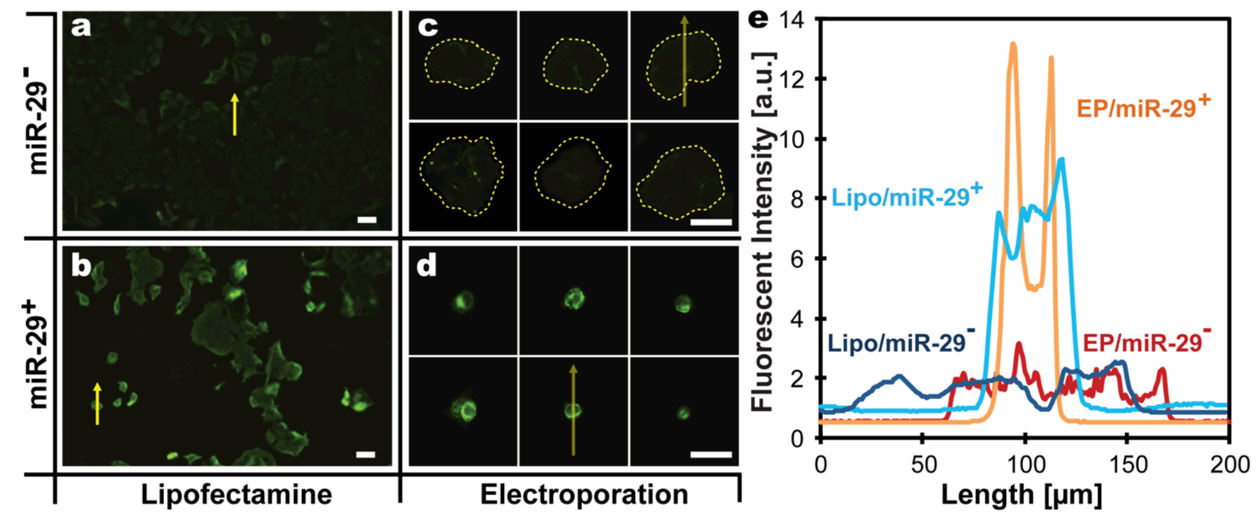

Supplementary Figure S6. Representative fluorescent microscopic images of MCF7 cells transfected via lipofectamine with **(a)** media or **(b)** miR-29, and via electroporation with **(c)** DPBS or **(d)** miR-29. Apoptotic cells were visualized and evaluated by staining with Annexin V conjugated with Alexa-488 48 hr post-transfection. Cells transfected with miR-29 exhibited an apoptotic phenotype, indicated by greater amount of externalized phosphatidylserine, than their blank$^-$ counterparts. Scale bars represent 50 μm. **(e)** Quantitative comparisons of intensity profiles for processed cells. The intensity profiles were obtained across cells marked with yellow arrows in (a – d).

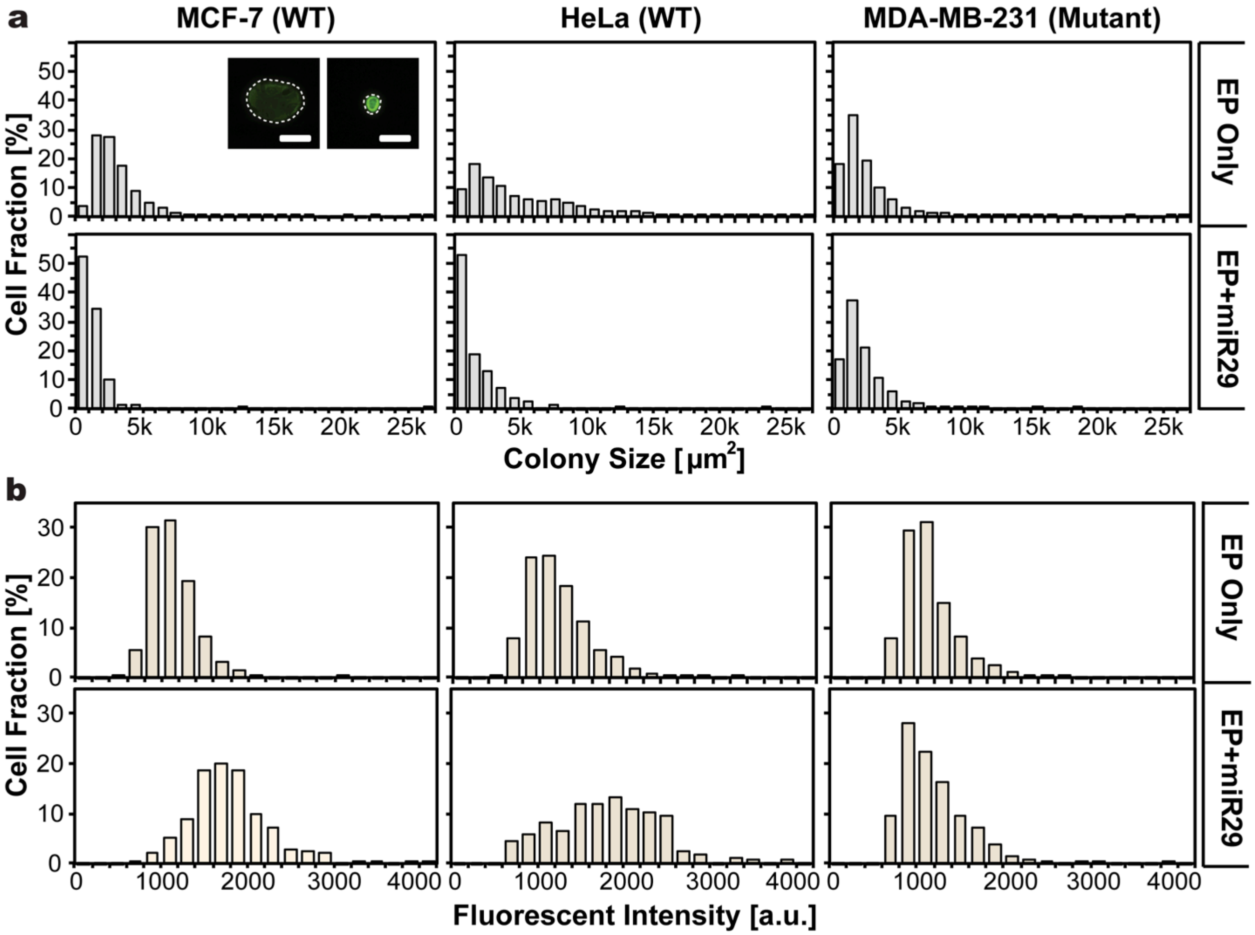

Supplementary Figure S7. **(a)** Colony size distributions for two p53 wildtype cell lines (MCF7 and HeLa) and p53 mutant cell line (MDA-MB-231) transfected with miR-29. Inset images illustrate how colony sizes were determined. Scale bars represent 50 µm. The decrease in colony sizes represents the morphological shrinkage of apoptotic cells. **(b)** Fluorescent intensity distributions of tested cell lines post Annexin V staining. The fluorescent intensity distributions were obtained within the region of interest (ROI) defined for the colony size measurements. The increase in fluorescent signals correlates with elevated levels of externalized phosphatidylserine, a phospholipid membrane component in the apoptotic cells. The mean fluorescent intensities of electroporated cell populations were calculated and compared in **Fig. 3b-c**. n = 648 (w/o) and 157; 1217 (w/o) and 337; 1142 (w/o) and 754 cells for MCF7, HeLa, and MDA-MB-231, respectively.

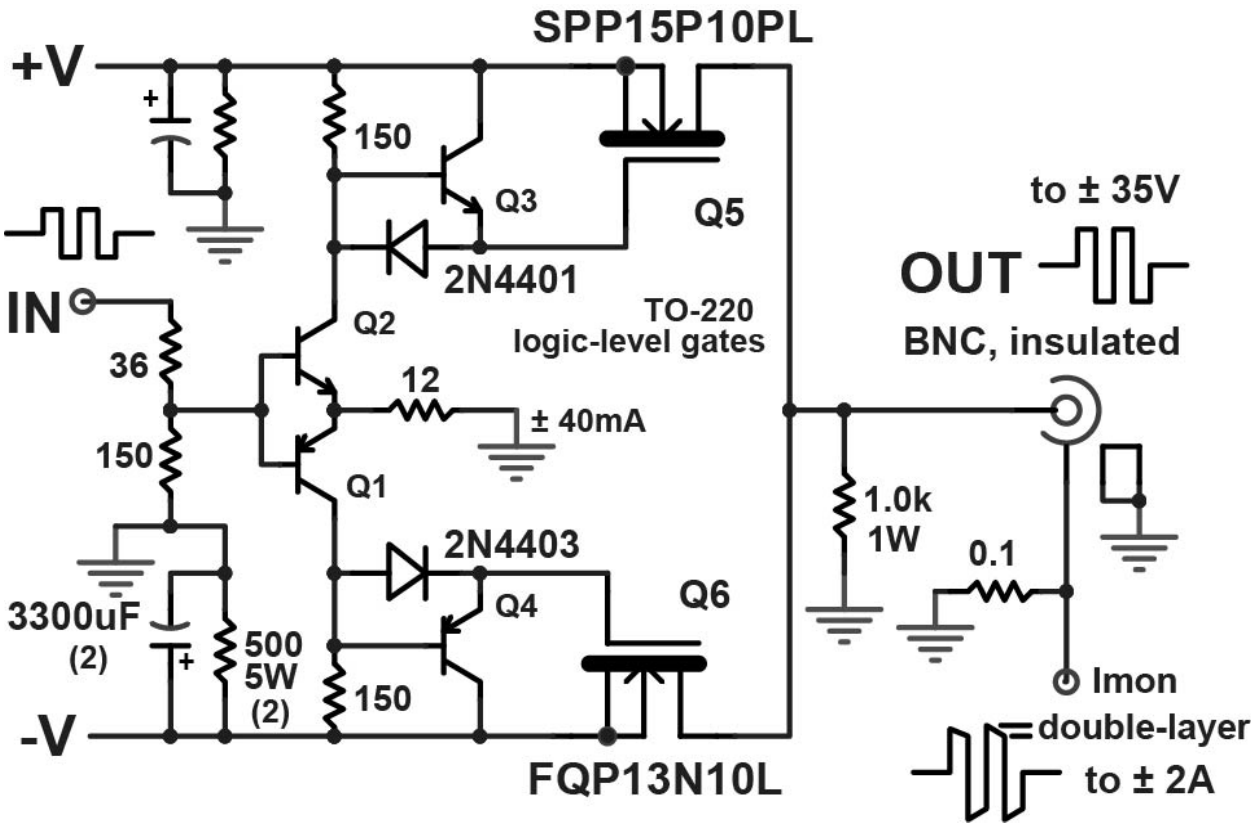

Supplementary Figure S8. A custom pulse driver was used to create programmable-amplitude bipolar voltage pulses for μSEA. The current of the system is monitored with a 0.1 Ω resistor.

Supplementary Table S1. Electrode layout and performance comparisons.

Performance Evaluation	Design 1	Design 2	Design 3[*]
**Voltage Efficiency[1] [%]**	76	90	80
**Cross-Chamber Voltage Variation[2] [%]**	23	2	5
**Total Chamber Number (row × chamber)**	22 (2×6+2×5)	16 (2×8)	40 (4×10)
**Chamber Structure (Width$_{\text{common bus line}}$ = 80 μm)**	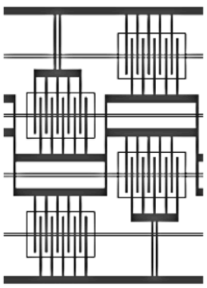	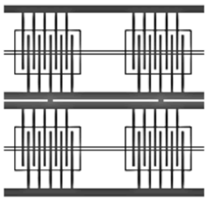	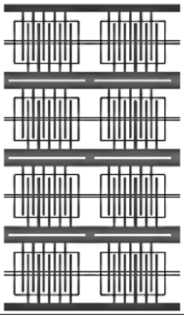

[*]Design 3 is chosen to be the final design of μSEA based on throughput (chamber number) and performance (voltage efficiency and cross-chamber voltage variation).

[1]Voltage efficiency = Voltage across the electroporation array / Applied voltage to the Au pads ×100%.

[2]Cross-chamber voltage variation = (Maximum chamber voltage - Average chamber voltage) / Average chamber voltage ×100%.
